# Nutritional Impact on Breast Cancer in Menopausal and Post-Menopausal Patients Treated with Aromatase Inhibitors

**DOI:** 10.3390/cancers18010073

**Published:** 2025-12-25

**Authors:** Roxana Popescu, Corina Flangea, Daliborca Cristina Vlad, Ionut Marcel Cobec, Peter Seropian, Cristina Doriana Marina, Tania Vlad, Andrei Luca Dumitrascu, Daniela Puscasiu

**Affiliations:** 1 ANAPATMOL Research Center, Department of Cell and Molecular Biology, Faculty of Medicine, “Victor Babes” University of Medicine and Pharmacy, 2nd Eftimie Murgu Square, 300041 Timisoara, Romania; popescu.roxana@umft.ro (R.P.); tania.vlad@umft.ro (T.V.); puscasiu.daniela@umft.ro (D.P.); 2 Department of Biochemistry and Pharmacology, Faculty of Medicine, “Victor Babes” University of Medicine and Pharmacy, 2nd Eftimie Murgu Square, 300041 Timisoara, Romania; flangea.corina@umft.ro (C.F.); cristina.marina@umft.ro (C.D.M.); 3 Department of Obstetrics and Gynecology, Faculty of Medicine, Medical Center-University of Freiburg, 79106 Freiburg, Germany; 4 Clinic of Obstetrics and Gynecology, Klinikum Freudenstadt, 72250 Freudenstadt, Germany; 5 Doctoral School, Faculty of Medicine, “Victor Babes” University of Medicine and Pharmacy, 2nd Eftimie Murgu Square, 300041 Timisoara, Romania; andrei.dumitrascu@umft.ro; 6 Intensive Care Unit Department, “Pius Brinzeu” County Emergency Hospital, Liviu Rebreanu Blvd. 156, 300723 Timisoara, Romania

**Keywords:** aromatase inhibitors, breast cancer, biologically active compounds, nutrients, drug interference

## Abstract

**Background/Objectives**: Aromatase inhibitors (AIs)—specifically, letrozole, anastrozole and exemestane—represent the current gold standard for patients with estrogen-receptor-positive breast cancer (ER + BC). This narrative review highlights potential interactions between nutrients and AIs, elucidating their molecular mechanisms involved. **Methods**: A comprehensive search was conducted across the PubMed, ScienceDirect, Google Scholar, and Scopus databases to identify scientific publications and elucidate recommended dietary regimes for ER + BC patients treated with AIs. **Results**: Certain bioactive substances found in licorice, rosemary, juniper, cannabis, and citrus fruits exhibit intrinsic aromatase-inhibiting effects. Additionally, other nutrients and compounds—including honey, ginger, turmeric, sweet potatoes, pomegranates, bitter melon, dark sweet cherries, resveratrol, and vitamins D and C—contribute to treatment outcomes through their demonstrated antiproliferative properties. Certain natural compounds, such as soy, cow’s milk, sesame seeds, and sesame oil, require caution due to their potential estrogen-like effects which could diminish the anti-estrogenic efficacy of AIs. **Conclusions**: These considerations hold significant weight in this context, as the management of oncological patients—particularly women with ER + BC—requires an integrated perspective. Antineoplastic treatment must be supported by appropriate nutrition to enhance antitumor efficacy and improve the patient’s quality of life. The data presented herein are derived from in vitro, in silico, and animal model studies and await validation in large patient cohorts. Nevertheless, these findings pave the way for future research to elucidate these molecular phenomena in humans and to establish clinically significant conclusions for ER + BC patients.

## Simple Summary

Aromatase inhibitors (AIs) are frequently used to treat menopausal or postmenopausal patients with estrogen-positive breast cancer. However, some cases exhibit primary resistance or develop secondary resistance to AI therapy. Nutrition appears to play a pivotal role in the patient’s journey from diagnosis onward, significantly influencing the clinical response to AI therapy. This review presents the interactions of various nutrients found in foods or dietary supplements, elucidating the molecular mechanisms by which Ais’ efficacy may be either attenuated or enhanced. In this context, alongside precisely targeted pharmacological therapy, the nutritional status of the oncological patient is of paramount importance. These considerations underscore the need for further research into expanding therapeutical options and developing personalized approaches that adapt to the changes occurring in the life of patient diagnosed with breast cancer.

## 1. Introduction

Breast cancer (BC) remains a major global health challenge, characterized by rising incidence rates, and is the subject of intensive worldwide research. Statistics indicate that the breast cancer is one of the most frequently diagnosed malignancies globally and remains the leading cause of death among the female population [1,2,3]. In addition, approximately 2.3 million new cases and 685,000 deaths were reported globally in 2020, representing a pressing public health concern [1,2]. According to a recent study, countries with a high human development index exhibit a higher incidence of BC but a lower mortality rates, primarily due to the implementation of screening programs, early diagnosis, and access to advanced treatments [4]. In contrast, countries with a lower human development index report significantly higher mortality rates [4]. According to current data, incidence rates are lowest in Africa and most of Asia, reaching intermediate levels in Eastern Europe and South America, whereas Australia, New Zealand, and most of Europe and North America report higher rates [5]. In terms of ethnicity and race, the highest incidences were recorded in White women, followed by Black women, and the lowest rates were observed in Asian/Pacific Islander, Hispanic, American Indian and Alaska Native women [6]. It is well-established that BC is a complex disease driven by a combination of external and internal factors. Its occurrence is rare before the age of 35, while the incidence increases significantly within the interval of 40–60 years of age [7,8,9]. The risk factors involved in BC development are categorized into non-modifiable factors such as BRCA1, BRCA2 genetic mutations, female sex, family history, and advanced age, as well as modifiable factors including obesity, smoking, alcohol consumption, physical inactivity, and an unhealthy diet [10,11,12]. Currently, BC treatment strategies encompass a wide range of options including a “less is more” surgical approach, radiotherapy, chemotherapy, endocrine therapy, and immunotherapy [13]. There is a growing recognition that nutritional status plays a critical role during BC treatment and recovery, significantly impacting the patient’s quality of life [14]. Patients diagnosed with BC often seek to optimize their nutritional habits by adopting specific nutritional regimes, using natural products or various vitamin and mineral supplements. Substantial evidence indicates that calcium and vitamin D supplements, along with natural products such as fish oil, omega 3-fatty acids, and turmeric, are frequently used as adjuvants by patients undergoing endocrine therapy [15]. Recent research indicates that certain adverse reactions to aromatase inhibitor (AI) therapy, such as osteoporosis, can be alleviated through lifestyle changes including regular physical activity, eating a balanced diet, and the supplementation of calcium and vitamin D [16]. Numerous patients adhere to the Mediterranean diet, which is considered an attractive concept due to its potential to reduce BC risk and, for those already diagnosed, to improve prognosis by decreasing the risk of recurrence [17]. The concept of the Mediterranean diet specifically refers to a high intake of vegetables, fruits, nuts, whole grains, and olive oil; a moderate consumption of fish, poultry, and dairy products; and limited intake of refined sugars and red meat [18,19]. Due to the high content of fibers, antioxidants, monosaturated acids, and polyunsaturated fatty acids, this dietary pattern is considered one of the healthiest; it exerts anti-inflammatory effects, increases insulin sensitivity, and reduces oxidative stress [20]. However, concerns have been raised about the potential of these dietary interventions and supplements to interact with medications within the therapeutic regimens.

Another study has also shown that obesity is a key factor correlated with disease progression, recurrence, and resistance to endocrine treatment [21]. Recent findings highlight that obese BC patients treated with AIs generally present with unfavorable outcomes, exhibiting higher rates of recurrence and mortality compared to those of non-obese patients [22].

Various spices, foods, dietary supplements, and plant extracts may potentially interfere with AIs. From a clinical perspective, one of the primary concerns for this patient population is “What am I allowed to eat and what should I avoid?” Beyond the standard contraindications against alcohol consumption and smoking, there is an ongoing debate regarding the interactions between AIs and diet. This includes the intake of complex food and supplements and whether they contain a single substance or a whole plant extract. In this narrative review, we discuss the recommended dietary regimen for patients with BC receiving AIs. These discussions, which sometimes border controversy, aim to elucidate the mechanisms of interactions from both a pharmacodynamic and pharmacokinetic point of view and to explain the potential benefits or disadvantages of certain natural products. This remains challenging because whole foods of natural origin consist of complex mixtures of bioactive substances, which can produce effects that differ from those of their isolated components. To this end, we have provided an overview of the currently established mechanism in the field, incorporating studies and expert opinions. Our objective is to identify scientifically justified solutions and dietary practices that are beneficial for BC patients undergoing AI treatment.

## 2. Methodology

This narrative review aims to present the current scientific evidence regarding the types of foods and supplements recommended for patients with BC undergoing AI therapy. To achieve this, we examine how AIs modulate estrogen production, the clinical consequences of these agents, and the effects of bioactive compounds from exogenous natural sources.

In the first section, we describe the role of aromatase in estrogen biosynthesis and provide the structural details of AIs widely used today, specifically, letrozole, anastrozole, and exemestane. For this description, the previously mentioned review articles have proven the most useful contributions to the conception of the initial schemes in the manuscript. In the second section, we examine the pharmacodynamic interactions between AIs and specific molecules introduced through dietary intake, alongside recent publications regarding their effects on ER or aromatase and their influences on AIs’ efficiency. Compounds with well-established and widely studied antiproliferative effects were also included. We searched for original articles and systematic reviews encompassing clinical and preclinical studies that demonstrate the described effects, and here, we outline the clinical consequences and explain their mechanisms of action. The composition and chemical structures of these nutrients were primarily documented from review articles. In the section dedicated to natural products that may inhibit the activity of AIs, we address the common clinical practice among oncologists/gynecologists of advising against the consumption of soy, milk, and sesame products. We present scientific evidence from various studies that either support or refute this practice. To ensure objectivity, we also examine pharmacokinetic factors, even though such interactions are not traditionally considered clinically relevant for AIs.

To accomplish this purpose, we used the scientific databases PubMed, ScienceDirect, Google Scholar, and Scopus using the following search terms: “breast cancer”, “aromatase”, “aromatase inhibitors”, “exemestane”, “anastrozole”, “letrozole”, “estrogen”, “*Humulus lupus*”, “*Glycyrrhiza*”, “citrus fruit”, “*Rosmarinus officinalis*”, “*Juniperus procera*”, “cannabidiol”, “*Curcuma longa*”, “*Zingiber officinale*”, “honey”, “sweet potato”, “*Punica granatum*”, “*Momordica charantia*”, “*Prunus avium*”, “resveratrol”, “vitamin D”, “vitamin C”, “soy”, “sesame”, “phatmacokinetics”, “*Hypericum perforatum*”, and various combinations among them. The search prioritized publications from the last 5 years. However, when data were insufficient—particularly in the final section or regarding clinical and preclinical trial results for key findings—we extended the search period back to 2015. We excluded non-peer-reviewed articles, conference abstracts, editorials, letters to the editor, and editorial notes. The search strategy was flexible, taking into account both specific mechanistic details and the information necessary for the introductory parts of each section and subsection.

## 3. Structural Considerations and Mechanisms of Action That Could Be Influenced by Natural Bioactive Compounds

### 3.1. Aromatase: Key Enzyme, Therapeutic Target of Aromatase Inhibitors

**Figure 1:**
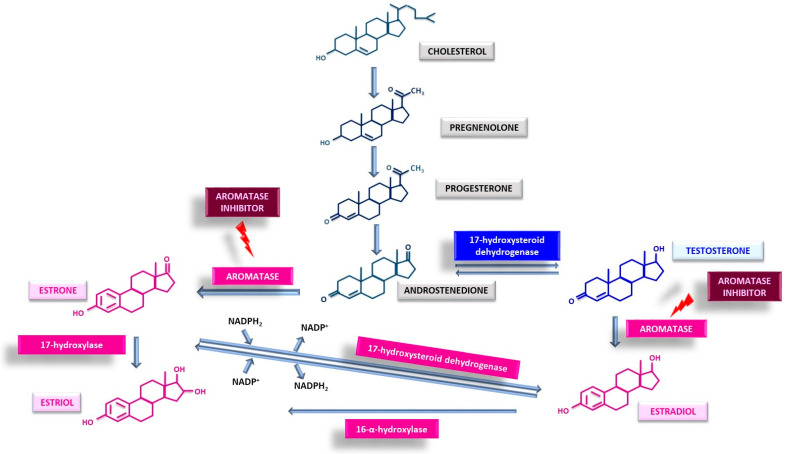
Schematic representation of estrogen synthesis; aromatase, the key enzyme, is displayed as the therapeutic target for aromatase inhibitors.

Estrogens are a class of steroid hormones with 18 carbon atoms synthesized in the human body comprising three active compounds: estradiol, the most potent and prevalent form, along with estriol and estrone [23,24]. They are produced by the ovaries with smaller quantities synthesized by the adrenal gland, placenta, liver, nervous system, and adipose tissue [25,26]. Estrogen biosynthesis originates from cholesterol and proceeds to androstenedione, following several transformations common to testosterone synthesis. From the androstenedione onward, aromatase plays a key role (Figure 1). In the initial stage of this common pathway, progesterone is formed through the dehydrogenation of pregnenolone followed by its conversion to androstenedione. Androstenedione can subsequently follow two metabolic paths: transformation into testosterone mediated by 17-hydroxysteroid dehydrogenase or conversion into estrone via the action of aromatase [27,28]. Estrone, in turn, will be converted either into estriol or estradiol, the latter of which can be subsequently transformed to estriol [29]. Thus, aromatase plays a crucial role in estrogen biosynthesis by acting on either testosterone or androstenedione, yielding the three primary estrogens which can undergo interconversion [30]. Consequently, the production of estrogens can be inhibited in the human body by targeting aromatase with AIs.

Once malignant transformation and proliferation occur in estrogen-receptor (ER)-positive BC, aromatase activity increases in correlation with the disease stage progression and the intensity of progesterone receptor expression [31]. Differences in aromatase activity may result from posttranslational modifications (PTMs) in the presence of BC, such as phosphorylation, glycosylation, acetylation, and ubiquitination. It has been demonstrated that PTMs might enhance the conversion of androstenedione to estrogens followed by increased aromatase expression in the presence of estradiol [32]. The process occurs in the context of ERα-mediated signaling, which is considered the primary driver of growth and proliferation of this type of BC [33]. In addition to the change in enzyme expression, mutations in the estrogen receptor 1 (ESR1) gene during malignant proliferation are further amplified in metastatic forms [34,35,36,37], being responsible for AI resistance [38,39,40]. This resistance develops over time, usually observed after 2–3 years of AI treatment [41]. In this context, the question arises of whether this resistance could be mitigated by incorporating a specific dietary pattern of the consumption of biologically active natural substances.

### 3.2. Aromatase Inhibitors: Structure and Properties

Following BC diagnosis and staging, the focus shifts to therapeutic strategies, with a growing interest in adjuvant and neoadjuvant treatments. It has been demonstrated that approximately 70% of invasive breast cancers are hormone-receptor-positive tumors. These respond to therapies that inhibit ER signaling pathways, including AIs (letrozole, anastrozole, and exemestane), selective ER modulators like tamoxifen, and ER down-regulators such as fulvestrant [42].

Tamoxifen was a breakthrough therapy in the management of breast cancer patients when it was introduced in the late 1960s and approved by U.S. Food and Drug Administration in 1977 [43,44]. In the current clinical landscape, there is a trend toward replacing tamoxifen with AIs in postmenopausal women. Furthermore, several clinical trials in premenopausal patients have indicated superior outcomes when combining aromatase inhibitors with ovarian function suppression (OFC), compared to that of tamoxifen plus OFC, or tamoxifen monotherapy [45]. Another aspect to consider is that approximately one-third of ER + BC cases develop tamoxifen resistance, which has a negative impact on the patient’s prognosis [46]. Consequently, alternative endocrine therapies must be evaluated [47]. Therefore, aromatase inhibitors are being increasingly explored as an alternative to tamoxifen in specific clinical scenarios. Although tamoxifen offered treatment advantages comparable to those of first- and second-generation aromatase inhibitors, the third-generation AIs have demonstrated superior efficacy [43]. Furthermore, endocrine treatment with aromatase inhibitors in postmenopausal women results in an additional proportional reduction in recurrence rates of approximately 30% [48]. Within the third generation of AIs, letrozole and anastrozole are non-steroidal compounds, while exemestane is a steroidal AI [49]. Some research comparing the third-generation aromatase inhibitors found that the best 5-year disease-free survival (DFS) was associated with letrozole, followed by exemestane, while the poorest outcome was observed with anastrozole [50].

**Figure 2:**
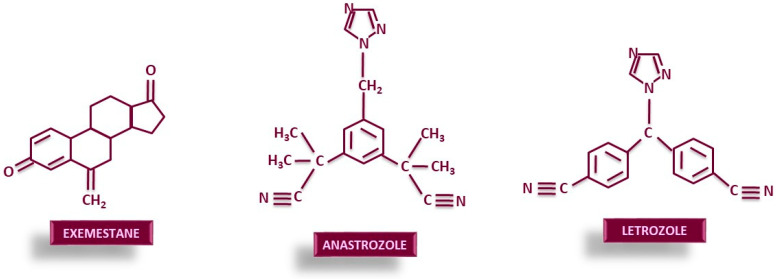
Chemical structures of AIs currently used.

Chemically, exemestane has a similar structure to the natural substrate, androstenedione (Figure 2). Both exemestane and its active metabolite 17β-dihydroexemestane irreversibly bind to the active site of aromatase [51]. In this way, aromatase remains inactivated even after the drug has been cleared from the body [52]. Letrozole and anastrozole cause a reversible competitive inhibition of aromatase by establishing non-covalent bonds with the heme group of aromatase. Structurally (Figure 2), both substances carry an imidazole ring [30,53,54]. Attachment occurs due to interaction with the iron within the heme core of aromatase [53].

Although AIs are generally well tolerated and widely used in hormone-receptor-positive BC, they are not entirely free of adverse effects [55]. Studies have shown an increased risk of cardiovascular events in patients treated with AIs [55,56]. Additionally, musculoskeletal adverse events such as arthralgias, myalgias, tendinopathies, and joint stiffness have also been reported in the literature [49,57]. Moreover, patients undergoing treatment with AIs may experience ophthalmic adverse effects, including dry eyes, decreased visual acuity, and retinal hemorrhage [58]. Among postmenopausal women with BC receiving AIs, symptoms such as vaginal dryness, decreased libido, and dyspareunia are frequently described; these affect the quality of sexual life and may therefore compromise treatment adherence [59]. Metabolic disruption, such as dyslipidemia, hyperglycemia, weight gain, and abdominal obesity, has been observed during AI therapy [60,61]. Furthermore, cognitive dysfunction is commonly associated with the depletion of estrogen resulting from this treatment [62]. This phenomenon is not apparent with exemestane during the first year of treatment [63]; however, the long-term impacts of these changes remain poorly characterized. Despite the potential of undesirable side effects, AIs remain the standard hormonal therapy for breast cancer patients. Additionally, third-generation non-steroid AIs are also utilized in endometriosis management to reduce pain-related symptoms [64].

## 4. Natural Products That Improve AI Action

Current trends in the treatment of cancer patients in general, and BC patients in particular, are moving towards the combination of dietary supplements or natural active principles with standard drug therapy as adjuvants. In this regard, various recent clinical studies have been conducted, as summarized in Table 1 for the period 2020–2025.

### 4.1. Biologically Active Plants and Compounds with Aromatase Inhibitor Effects

**Figure 3:**
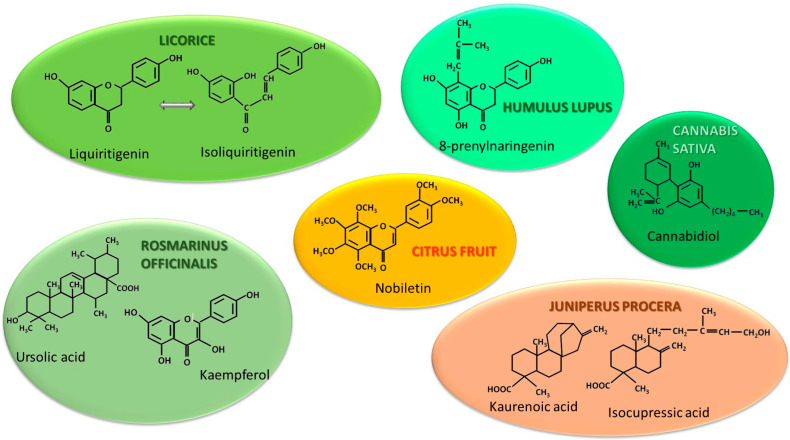
Structures of the most important biologically active compounds for aromatase inhibitory action.

Certain natural compounds exhibit inhibitory effects on aromatase. Among them, several phytoestrogens stand out for their competitive and reversible inhibitory mechanism, while other natural compounds may exert non-competitive inhibition. These substances, along with the studies highlighting their effects, are described below and illustrated in Figure 3.

#### 4.1.1. Hops of *Humulus lupulus* (HHL) and *Glycyrrhiza* or Licorice Species

Hops of *Humulus lupulus* (HHL) and *Glycyrrhiza* or licorice species contain a series of phytoestrogens that competitively inhibit aromatase in a similar manner to that of letrozole [71]. Several structures stand out in this regard: 6-prenylnaringenin, xanthohumol-isoxanthohumol pair, which subsequently converted to 8-prenylnaringenin found in hops, and licochalcone A, 8-prenylapigenin, isoliquiritigenin-liquiritigenin pair identified in licorice [71,72]. Among these substances, liquiritigenin appears to be the most promising for antitumor activity. Its efficacy was demonstrated through in silico [72,73] and in vivo studies on tumor tissue that had undergone unilateral or bilateral mastectomy [72,74] as well as on athymic nude mice inoculated with BT-474 breast cancer cell line (ERα+, derived from ductal carcinoma) [74]. This effect is further amplified by the capacity of liquiritigenin to reduce tumor invasiveness by upregulating E-cadherin and downregulating N-cadherin expressions. Furthermore, it triggers apoptosis by increasing caspase 3 activity [75]. Another advantage of these compounds is the delay in osteoporosis onset for estrogen-depleted women, an effect induced by HHL phytoestrogens [76]. Other studies highlight the anti-carcinogenic effect of xanthohumol and 6-prenylnaringenin, which act by increasing the production of reactive oxygen species (ROS) and triggering apoptosis in BC cells [77,78]. The potent anti-tumor effect of xanthohumol on BC cells has been further confirmed by meta-analysis [79]. Conversely, some authors suggest that HHL should be avoided in the presence of BC or other estrogen-dependent cancers, as phytoestrogens (especially 8-prenylnaringenin and 6-prenylnaringenin) act as agonists on both ER-α and ER-β [80,81]. The findings highlight a heterogeneity of the reported data; however, multiple clinical studies showing the results on humans are still lacking.

#### 4.1.2. Citrus Fruits, Including Their Peel

Citrus fruits, including their peel, are a source of flavonoids, among which the following substances stand out for their inhibitory effect on aromatase in a similar manner to letrozole: naringenin, quercetin, naringin [82], hesperidin [83], and nobiletin [84]. This property has been demonstrated by in silico studies and validated on MCF-7 BC cell lines (ERα+) derived from metastatic carcinoma both directly or after inoculation into athymic mice [82,83,84]. In brief, naringenin and quercetin interact with the aromatase hydrophobic catalytic domain specifically at the sterol-binding site. This is evidenced in vivo by a decrease in aromatase levels of 72.35% for quercetin, 62.43% for naringenin, and 59.36% for naringin [82]. In the case of nobiletin [84], aromatase activity in the MCF-7 cell line was inhibited only at low concentrations, specifically, 0.1 µmol/L. Although these findings are notable, the combination of letrozole with nobiletin showed no effect on aromatase activity [84]. One explanation may be the competitive mutual inhibition of these two substances, as they both have the same mechanism of action. These effects have only been observed in preclinical investigations. Consequently, large clinical trials are needed to elucidate this aspect.

#### 4.1.3. *Rosmarinus officinalis* (RO)

*Rosmarinus officinalis* (RO) is an aromatic plant commonly used as a spice in many culinary preparations. It possesses antimicrobial, antioxidant, antidiabetic properties, and is also known to promote recovery from skin injuries and wound healing [85,86,87,88]. Moreover, cytotoxic activity against malignant tumors including glioblastoma, rhabdomyosarcoma [89], epithelial carcinoma [90], colorectal carcinoma [91], non-small-cell lung cancer [92], and triple-negative BC [93] has been identified. RO contains two compounds that inhibit aromatase in a dose-dependent manner: ursolic acid and kaempferol [94]. Due to its structural similarity to androstenedione, ursolic acid appears to attach to the sterol derivative binding site of aromatase through hydrophobic interactions [94]. Another study demonstrated that ursolic acid possesses AI properties similar to those of exemestane and letrozole; however, its downregulation of the enzyme is attributed to the silencing of aromatase gene transcription [95]. Unlike ursolic acid, kaempferol acts directly on the catalytic center of the enzyme [94], suggesting a competitive mechanism. Aromatase inhibition elicited by kaempferol is relatively modest, reaching 30% at a concentration of 10 µM and 50% at 50 µM [94]. In contrast, ursolic acid demonstrated a tumor-suppressing index of 90% after 6 weeks of treatment in an MKN45 gastric cancer xenograft, where prognosis was directly correlated with the degree of aromatase expression [95]. Other natural sources of ursolic acid are thyme, oregano, marjoram, apple peels, and lavender [96,97,98]. These findings position ursolic acid a promising agent in the management of BC and establish a foundation for transitioning from in vitro studies, in silico studies, and animal models to clinical trials that could elucidate its role in BC patients treated with AIs.

#### 4.1.4. *Juniperus procera* (JP)

*Juniperus procera* (JP) is a plant used in traditional medicine for its antimicrobial, antifungal, antitumor, and antioxidant properties [99,100] as well as for its uses in treating digestive, liver, gout, and jaundice disorders [101,102]. In ovarian cancer research, JP exhibits high antioxidant activity and inhibition of topoisomerase II A, as illustrated by an in silico analysis [103]. Several studies using docking investigations have demonstrated that certain compounds extracted from JP possessed competitive inhibitory activity on aromatase: 2-imino-6-nitro-2H-1-benzopyran-3-carbothiamide [104], juniperolide, kaurenoic acid, and isocupressic acid [105]. Another study observed that the extract from fruits suppresses the aromatase-encoding gene in the MCF-7 breast cancer cell line. Conversely, under the same experimental conditions, it induces aromatase activation in the triple-negative MDA-MB-231 cell line (often use as a model for advanced BC) [106]. Given these findings, JP fruit extract or the fruits themselves may serve as a potential adjuvant dietary supplement for BC patients. In addition, the hypothesis that the transformation of an aggressive histological form into a less aggressive one could serve as a starting point in further research to establish the conditions under which this occurs in humans. At this stage, the possibility of pharmacological exploration of JP-derived compounds opens up with the aim of developing new therapeutic compounds.

#### 4.1.5. Cannabinoids

Cannabinoids, especially cannabidiol (CBD), the non-psychoactive component of cannabis sativa, have numerous therapeutic effects: antidepressant, anxiolytic, anti-inflammatory, and tissue regeneration [107,108,109]. Being anti-inflammatory, CBD causes a reduction in IL-6 levels [110] and inhibition of COX-2 [111]. In cancer management, CBD can be used for its cytotoxic and antiproliferative effects, including cell cycle arrest and induction of apoptosis [112,113,114]. Furthermore, it is effective in analgesic therapy for pain management [115,116]. CBD has demonstrated a competitive inhibition of aromatase in MCF-7 cell lines; however, its combination with anastrozole and letrozole did not indicate additional therapeutic benefits. In contrast, combining CBD with exemestane could be a beneficial solution in advanced stages. Some authors have identified high concentrations of estradiol in the cellular environment of early-stage BC tissue, where CBD could interact competitively on enzymatic active sites [117]. In silico analysis performed by the same group of authors supports these arguments [118]. Other researchers highlight the benefits of combining CBD with AIs in patients with hormone-receptor-positive breast cancer. This approach aims to reduce inflammation and pain associated with AI-induced musculoskeletal symptoms as well as to alleviate anxiety and insomnia. This phase 2 clinical trial included 39 patients with BC treated with standard doses of AIs (1 mg anastrozole, 25 mg exemestane, or 2.5 mg letrozole) who received up to 100 mg of CBD as the commercial preparation Epidiolex for 15 weeks [65]. Beyond the advantages related to side effect reduction, further clinical trials and extended studies on optimal dosing and drug–drug interactions are necessary to validate these experimental findings.

In general, research on the mechanisms by which certain substances inhibit the key enzyme of estrogen synthesis is performed in silico and in vitro. In a study involving a large number of commercially available organic compounds that may act as bioactive components in some natural products, their effects on aromatase were investigated through in silico and in vitro analysis using the MCF-7 cell line. The authors identified competitive inhibition exerted by chrysin, apigenin, and resveratrol in concentration ranges of 1.7–15.8 µM. In contrast, pomiferin and berberine exhibit non-competitive inhibition at concentrations of 15.1 µM and 21.4 µM, respectively [119]. When a single dietary supplement is considered, conclusions are evaluated individually; however, when complex natural products are discussed, the assessments are multifaceted, and overall results may vary. Moreover, species that act through a non-competitive mechanism or by downregulating aromatase gene transcription could serve as effective adjuvants for this patient category. In conclusion, bioactive substances that function as competitive aromatase inhibitors require precautions until future investigations definitively clarify these issues.

### 4.2. Biologically Active Plants and Compounds with Indirect, Antiproliferative Action

Certain plants or dietary supplements contain bioactive substances with broad antiproliferative properties, making them suitable as adjunctive therapies for different types of cancer. Most of these compounds have a well-established role in preventing the onset of malignant transformation. The most frequently used botanicals or dietary supplements from around the world utilized by ER + BC patients undergoing AI treatment are briefly characterized below.

#### 4.2.1. The Ginger Family

The ginger family, Zingiberaceae, includes two well-known members used as culinary spices: *Curcuma longa* with its product, turmeric, and *Zingiber officinale*, or ginger [120,121,122,123,124,125]. The antiproliferative effect of turmeric in BC has been demonstrated in MCF-7 and MDA-MB-231 cell lines [126,127]. Briefly, the Janus kinase (JAK)/signal transducer and activator of transcription (STAT)/phosphatidylinositol-3 kinase(PI3k)/protein kinase B (AkT)/mammalian target of rapamycin (mTOR) signaling pathways are overreactive in BC, driving tumor cells’ survival and proliferation [128,129]. This pathway is activated simultaneously by proinflammatory cytokines [130,131], epidermal growth factor (EGFR) [132,133], and vascular endothelial growth factor (VEGF) [130]. This activation promotes protein synthesis, cell proliferation, and growth, as well as cell cycle progression, while inhibiting apoptosis (especially via intrinsic pathway) to ensure cell survival [128,129,130,131,132,133]. One of the regulatory factors of this pathway is AMP-activated protein kinase (AMPK), which exerts an inhibitory effect on PI3k/AkT/mTOR [134]. Apoptosis can be suppressed through AkT-mediated inhibition of caspase 9 and the activity of histone deacetylases (HDAC) or by modulating the regulatory factors such as P53 and P21, along with the downregulation of pro-apoptotic proteins [135]. AkT promotes cell cycle progression by upregulating cyclins D1 and E1 and cyclin-dependent kinases (CDK) 2 and 4, while downregulating P21 [136]. Similar effects are mediated by nuclear factor-kB (NF-kB), specifically through its upregulation of cyclin D1 [137]. AkT can upregulate mTOR signaling, contributing to angiogenesis, cell proliferation, and survival through the mechanistic target of rapamycin complex 1 (mTORC1) and complex 2 (mTORC2) [138]. Curcumin, the primary active compound, is able to inhibit the EGFR pathway [139] and the initiation of PI3k/Aktcascade. This effect supports its use as an adjuvant in the treatment regimens of patients with BC [140]. Furthermore, curcumin can cause cell cycle arrest at the G1/S transition by reducing expressions of cyclins D1, E1, CDKs 4 and 2, and in the G2/M phase in MCF-7 cells [141]. In addition, curcumin has been shown to reduce elevated expressions of HDAC 1 and HDAC2 in MCF-7 and MDA-MB-231 cell lines, promoting cell cycle arrest through P21 upregulation of and the stimulation of apoptosis [142]. Other effects of curcumin include its role in weight loss by activating thermogenesis and inducing white adipose tissue browning [120,143], processes that are particularly desirable in obese BC patients. In a randomized double-blind placebo-controlled clinical trial involving 34 patients, the administration of 200 mg/day curcumin nanoemulsion in combination with AIs was evaluated over a three-month period [66]. Curcumin demonstrated a contribution to the improvement of joint pain and inflammation. No pharmacokinetic or pharmacodynamic interactions with AIs were observed, as assessed by monitoring plasma estrogen levels. The short duration of the study did not reveal whether curcumin exerts any antiproliferative contribution in enrolled patients. Clinically, inhibition of the PI3k/AkT pathway contributes to the reduced proliferation and metastasis [144]. Furthermore, this inhibition may sensitize cancer cells to biological therapies targeting the EGFR pathway [145]. Additionally, curcumin’s role in mitigating the osteoarticular adverse effects of AIs through anti-inflammatory action should not be overlooked [66].

Among the compounds in ginger, gingerols reduce BC invasion and metastasis by inhibiting CDKs and cyclins as well as suppressing Akt activation in the MDA-MB-231 cell line [146]. Gingerenone attenuates the cell cycle in MCF-7 and MDA-MB-231 cells [147], while shogaol is able to reduce the activity of HDACs [148]. Gingerol is generally an antioxidant that modulates the release of adipokines in adipocytes [149] and contributes to the reduction of hyperglycemia in patients with type 2 diabetes [150], while shogaol has predominantly antitumor activities [151,152].

#### 4.2.2. Honey

Honey is a natural product that contains several biologically active polyphenols. Among honey polyphenols, pinocembrin and chrysin exhibit antioxidant activity mainly by stimulating superoxide dismutase activity and reducing malondialdehyde [153,154,155], while caffeic acid inhibits lipid peroxidation with a protective effect on cell membranes [153,156,157]. The broad antiproliferative properties of honey results from a combination of numerous actions upon tumor cells, such as cell cycle arrest, apoptosis stimulation, antioxidant and anti-inflammatory action. These effects have been highlighted and discussed in several recent reviews [153,158,159,160,161], some of which focus specifically on breast cancer [162,163,164]. Recently, Marquez-Garban et al. [165] demonstrated that Manuka honey effectively reduces the proliferation of MCF-7 tumor cells although the antiproliferative effect was less pronounced in MDA-MB-231 cell lines. This action is due to the AMPK activation. It seems that polyphenols, especially caffeic acid, chrysin [165,166,167], and pinocembrin [168], are responsible for this effect. In a migration scratch assay, coniferous honeydew honeys in a concentration 0.1%v/v revealed a migration of around 65% in the MCF-7 cell line [169]. The inhibition of proliferation and migration as demonstrated by scratch assay in cells treated with Ziziphus jujube honey was attributed to the induction of apoptosis, characterized by the overexpression of P53, P21 and an increase in the Bax/Bcl2 ratio [170]. Tualang honey produced similar results on MCF-7 cells on P53, P21 expressions and activated caspases 3, 7, and 9 [171]. These effects are prominent in natural honey, whereas commercial honey exhibited significantly lower quality [172], particularly regarding its impact on the MCF-7 cell line [170]. Clinically, the activation of AMPK by honey may improve wound healing and tissue repair [173], while providing anti-inflammatory [173,174], antioxidant, mitochondrial [173,175], and cardiovascular protection [173,175]. These effects are beneficial in oncology, as they could mitigate the adverse effects on soft tissues and enhance post-surgery recovery. Furthermore, these protective properties are significant given that many chemotherapeutic agents are known to be cardiotoxic.

#### 4.2.3. Sweet Potato (SP)

Sweet potato (SP) contains several biologically active substances; among these, lipid-soluble polyphenols (PPLs) such as chlorogenic acids (CA), caffeic acid and its esters, exhibit potent antioxidant and antiproliferative properties [176]. In addition, SP anthocyanins in association with polyphenols can normalize blood glucose in type 2 diabetes [177,178]. In a study conducted on C57BL/6N mice inoculated with murine breast cancer cells E0771 (BC epithelial-like cells) and on the MDA-MB-231 cell line, PPLs demonstrated cytoplasmatic accumulation and reduced Akt phosphorylation at serine 473, leading to cell cycle arrest in the G0/G1 phase [179]. Treatment with SP-derived CA in nude mice inoculated with MB231 and MCF-7 cell lines via the tail vein resulted in reduced tumor migration and invasion. Following two months of intragastric administration of purified CA extract at a dose of 50 mg/kg/day, a significant decrease in the size of lung metastases was observed [180]. Carotenoids, another group of lipid-soluble substances, are found in SP, the most studied being lutein, zeaxanthin, β-cryptoxanthin, β-carotene, and α-carotene. They have demonstrated several antiproliferative effects on the MCF-7 cell line, including cell cycle arrest in the G0/G1 phase and increased activity of caspases 3, 8, and 9 [181]. Among the anthocyanins identified in SP, cyanidin is the primary compound exhibiting an antitumor effect in Rattus norvegicus models of BC; this is attributed to inhibition of the JAK/STAT and VEGF pathways [182]. Inhibitory effects on tumor growth were also observed in the BT549BC cell line (triple-negative, derived from invasive ductal BC) following 24 h exposure to high concentrations (0.00313 mg/mL) of SP leaf extracts. The authors attributed this result to the presence of anthocyanins and polyphenols [183]. Clinically, inhibition of the VEGF and JAK/STAT pathways results in a reduction in angiogenesis and tumor proliferation while promoting apoptosis [181].

#### 4.2.4. *Punica granatum* (PG) or Pomegranate

*Punica granatum* (PG) or pomegranate peel and juice contain several substances with antiproliferative activity such as ellagitannins (punicalin, punicalagin, casuarinin, gallagildilactone, pedunculagin, tellimagradin, corilagin, granatin A, and granatin B), anthocyanins (cyanidin, delphinidin, and pelargonidin), and polyphenols. Among these, flavonoids (catechin, epicatechin, epigallocatechin-3-gallate, quercetin, rutin, kaempferol, luteonin, and naringenin) and hydroxybenzoic acids (gallic acid, ellagic acid, caffeic acid, and chlorogenic acid) are the most abundant [184,185,186]. These substances are responsible for several biological properties, including antifungal, antimicrobial, and anti-inflammatory. The latter is characterized by decreased COX2 expression and the reduction in PGE2, nitric oxide synthesis, alongside potent antitumor effects [187,188]. The composition, concentration, and biological effect of the aforementioned compounds on BC cells vary depending on the color of the fruit [189]. Incubation of MDA-MB-231 cells with 10% PG juice results in significant reduction in cell proliferation after 48 h [190]. Furthermore, PG extract can induce cell cycle arrest and apoptosis in breast cancer tumor cells [185,191,192] without affecting normal, non-malignant cells [193]. Punicalin and punicalagin nano-prototypes exert cytotoxic and apoptotic effects on breast MCF-7 and MDA-MB-231 cancer cell lines by increasing caspase 3 expression and the Bax/Bcl2 ratio [194]. Through this mechanism, PG extract can sensitize tumor cells to the effects of chemotherapeutic agents [195]. In addition, punicalagin inhibits migration in the same cell lines by downregulating N-cadherin and upregulating E-cadherin, thereby preventing tumor dissemination and metastasis [185,196].

#### 4.2.5. *Momordica charantia* (MC) or Bitter Melon

*Momordica charantia* (MC) or bitter melon: MC is widely used as a dietary supplement in the treatment of type 2 diabetes mellitus due to its ability to inhibit glucose uptake [197,198,199,200]. Recently, MC demonstrated significant antitumor properties in breast cancer models [201,202,203]. In a study where MCF-7 cells were incubated with an MC extract, a significant cytotoxic effect was observed; this was attributed to the inhibition of glucose uptake [204]. MC is a rich source of triterpenes and terpenoids including cucurbitanes, momordicin, momordicosides, karaviagenins, karavilosides, charantins, limonene, and lupenone. It also contains polyphenols such as kaempferol and quercetin [199,200,205]. Among the compounds studied, some authors identify kaempferol, quercetin, and momordicoside K as the most promising owing to their potential to inhibit STAT3 in silico [199]. Other authors have shown that the triterpenoid 3β7β25-trihydroxycucurbita-5,23(E)-dien-19-al induced apoptosis in MCF-7 and MDA-MB-231 cells by activating the peroxisome proliferator-activated receptor γ (PPARγ) pathway and P53 phosphorylation while inhibiting NF-kB signaling [206]. Another study on MCF-7 and MDA-MB-231 cell lines highlights that MC extract induces autophagy via activation of the AMPK signaling pathway [207]. Regarding the clinical response, in addition to inhibiting abnormal cell growth, metabolic balance is restored by increasing glucose uptake and promoting fatty acid oxidation [208]. Furthermore, MC extract demonstrated an inhibitory effect on the production of proinflammatory molecules such as IL-1β, IL-6, and TNF-α in the aforementioned cell lines and in other BC models, thereby decreasing inflammatory signaling [209,210].

#### 4.2.6. *Prunus avium* (PA) or Dark Sweet Cherry

*Prunus avium* (PA) or dark sweet cherry: PA possesses a rich content of polyphenols and anthocyanins. PA components demonstrated the ability to reduce lipid peroxidation and decrease LDL oxidation [211]. Additionally, other identified effects of PA fruits include improved memory, increased information processing speed, and enhanced cognitive performance [212]. The most important active molecules—namely, cyanidin-3-O-rutinoside, cyanidin-3-glucoside, and peonidin-3-O-rutoside [213]—are considered such for their increased efficacy against advanced BC [213,214,215,216,217]. Anthocyanins suppress the Akt/mTOR signaling pathway in both 4T1BC [214] and MDA-MB-231 cell lines [218]. When anthocyanins extracted from PA are co-administered with doxorubicin for 48 h, a synergistic effect is observed in 4T1BC cell lines [215] and in metastases induced by this cell line in female BALB/C mice [213], thereby supporting the efficacy of conventional chemotherapy. In patients with non-metastatic BC treated with AIs, tart cherry extract was found to alleviate treatment-induced arthralgia [219].

#### 4.2.7. Resveratrol

Resveratrol, a polyphenol found mainly in grapes but also in some berries, has antiproliferative, antioxidant, neuroprotective, anti-inflammatory, antidiabetic, and cardioprotective properties [220,221,222,223]. Although primarily recognized for its anti-aging properties [224,225], resveratrol is currently being intensively studied for its antiproliferative effect. It occurs naturally as the trans-isomer which can isomerize to the cis form upon exposure to UV radiation [226]. In BC, its antitumor activity is mediated by several mechanisms. Resveratrol decreases STAT3 activity by reducing acetylation at the LYS685 residue, which triggers ESR1 activation via demethylation. This process restores sensitivity to antiestrogen therapy [227]. Additionally, ESR1 activation is associated with enhanced osteogenesis and a partial reduction of bone resorption [228]. Incubation of 4T1BC and MDA-MB-231 cell lines with triphenyl phosphonium-conjugated resveratrol induces a loss of mitochondrial membrane potential, mitochondrial swelling, and disruption of purine and pyrimidine metabolism, ultimately leading to apoptosis [229]. Another study investigated the delivery of resveratrol and tamoxifen using lipid-based and liquid crystalline nanoparticle systems in MCF-7 cells. Malignant cells treated in this manner showed, after 24 h, enhanced caspase 8 activity. This approach also resulted in reduced tumor cell viability and a significant mitigation of tamoxifen’s toxicity to normal cells (ATCC CL-48). From a clinical perspective, a sensitization of BC cells to chemotherapy and a modulation of the tumor environment were observed [230]. Moreover, resveratrol-loaded chitosan nanoparticles stimulated autophagy in MDA-MB-231 cells, as evidenced by the upregulation of the autophagic genes Beclin-1, ATG-5, ATG-7, LC3A, and P62 after 48 h of incubation [231]. Metabolically, resveratrol reduces ATP production in tumor cells by interfering with both carbohydrate and lipid metabolism. Thus, resveratrol has been shown to inhibit phosphoglycerate kinase 1 (a key enzyme in the Meyerhoff–Embden pathway) in BT-549 cells, thereby blocking glycolysis [232]. Simultaneously, fatty acid β-oxidation is inactivated through the alteration of the carnitine/acylcarnitine fatty acid transport system across the mitochondrial membrane in MCF-7 and MDA-MB-231 cells [233]. Furthermore, resveratrol inhibits fatty acid synthase, which is usually overexpressed in BC [234].

#### 4.2.8. Vitamin D

Vitamin D deficiency, specifically, low levels of 25-hydroxyvitamin D, is a common feature observed in patients with BC [235,236]. The primary biological role is played by calcitriol (1,25-dihydroxyvitamin D3), the active metabolite with the most potent biological activity. The co-administration of calcitriol with AIs contributes to a reduction in tumor proliferation in the MCF-7 cell line [235] by downregulating ERα mRNA expression [237]. This downregulation of ERα induced by the concomitant administration of calcitriol and the 1,24-dihydroxyvitamin D3 derivative enhanced the efficacy of anastrozole in mice with MCF-7 induced BC [238]. On the other hand, since BC patients treated with AIs face an increased risk of developing osteoporosis, vitamin D supplementation is a routine practice in these cases [239,240]. Another benefit of vitamin D observed in BC patients is the reduced expression of proinflammatory markers IL-6 and TNFα alongside an increase in the IL-10/TNFα ratio. These effects were significant at doses of 4000 IU/day but were not observed at a dose of 2000 IU/day [241]. When a probiotic mixture of *Lactobacillus* strains (*L. casei*, *L. acidophilus*, *L. rhamnosus*, *L. salivarius*, and *L. reuteri*) and *Bifidobacterium* strains (*B. lactis*, *B. longum*, and *B. bifidum*) is administered in combination with vitamin D at 1000 IU/day, a significant increase in the anti-inflammatory index is observed. In contrast, vitamin D monotherapy at the same dose fails to produce detectable changes [242].

#### 4.2.9. Vitamin C

Vitamin C is utilized as a complementary therapy in BC, exhibiting antiproliferative effects at high doses against MCF-7 and MDA-MB-231 cell lines resistant to chemotherapy and hormone therapy (e.g., docetaxel, doxorubicin, and tamoxifen) [243]. Evidence suggests that high doses of vitamin C inhibit NF-kB, thereby sensitizing BC cells to hormone therapy, chemotherapy, and radiotherapy [244]. In one study, vitamin C was administered intravenously to a cohort of 350 patients with BC undergoing concurrent hormone, chemotherapy, and radiation treatments. At a dosage of 25 g/week for four weeks, an improvement in quality of life was observed specifically through a reduction in the severity of vomiting, pain, fatigue, and appetite loss [245]. Furthermore, the co-administration of vitamin C with BC-specific treatment led to an improvement in overall survival (OS), a finding highlighted by a recent systematic review [246].

**Figure 4:**
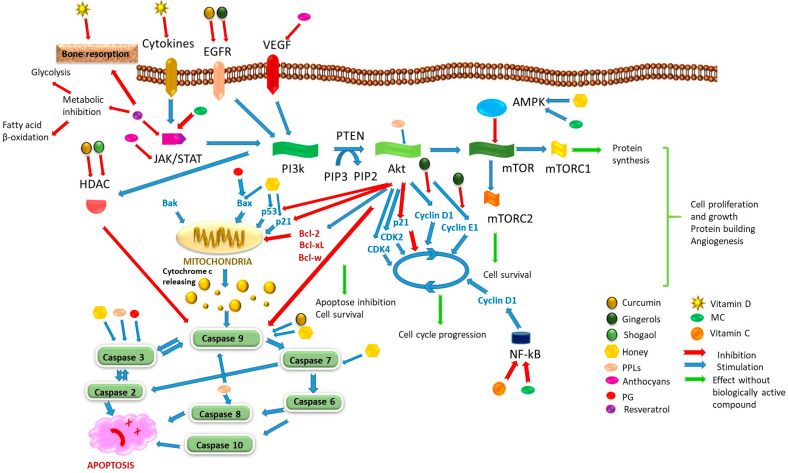
Summary of the main mechanisms modulated by certain natural substances including the nature and location of their molecular influence on these cell signaling pathways. The biological effects ilustrated represent those occuring in the absence of biologically active compounds. The intervention sites are marked as stimulatory (blue arrow) or inhibitory (red arrow). Natural compounds modify these responses by inducing apoptosis, cell cycle arrest, and the inhibition of angiogenesis protein synthesis, ultimately leading to cancer cell death. (EGFR—epidermal growth factor receptor; VEGF—vascular endothelial growth factor; HDAC—histone deacetylases; JAK—Janus kinase; STAT—signal transducer and activator of transcription; PTEN—phosphatase and tensin homolog; PIP3—phosphatidylinositol triphosphate; PIP2—phosphatidylinositol bisphosphate; AkT—protein kinase B; AMPK—AMP-activated protein kinase; PI3k—phosphatidylinositol-3 kinase; mTOR—mammalian target of rapamycin; mTORC—mechanicistic target of rapamicyn complex; NF-kB—nuclear factor-kB; CDK—cyclin-dependent kinase).

In conclusion, the indirect antiproliferative effects of certain biologically active natural compounds are mediated by the modulation of specific signaling pathways. Activation of PI3k/Akt/mTOR and its related pathways, which is frequently upregulated in cancer cells, leads to cell growth, proliferation, protein synthesis, and angiogenesis, while simultaneously inhibiting apoptosis. Consequently, most of these compounds induce apoptosis and trigger cell cycle arrest, thereby reducing the viability of BC cells. Furthermore, they possess the potential to inhibit angiogenesis. These effects are mediated through both cellular and extracellular mechanisms, such as the reduction of proinflammatory cytokines by MC and vitamin D. It should be noted that these findings have been demonstrated exclusively in cell cultures and animal models. Figure 4 summarizes these mechanisms alongside the described biological effects and modulatory influences of natural bioactive compounds. These natural extracts demonstrate potent antitumor activity, particularly when used in high concentrations or as a complex formulation. Although in vitro or in vivo studies in laboratory animals are conclusive, long-term results in humans are not so satisfactory. Further research is needed to improve the bioavailability of these natural compounds and the benefits of their maximum potential observed in lab experiments.

## 5. Natural Products That Inhibit AI Action

### 5.1. Estrogen-like Natural Compounds

Estrogen-like natural compounds are biological active substances with structures similar to those of endogenous estrogens, enabling them to bind to ER and produce a specific stimulatory response. When AIs are used, endogenous estrogen synthesis is stopped; however, the exogenous intake of estrogen mimetics could theoretically contribute to the therapeutic failure of hormone therapy in ER + BC patients. Understanding the sources of these compounds is crucial for the overall therapeutic outcome in this patient population.

#### 5.1.1. Soy

**Figure 5:**
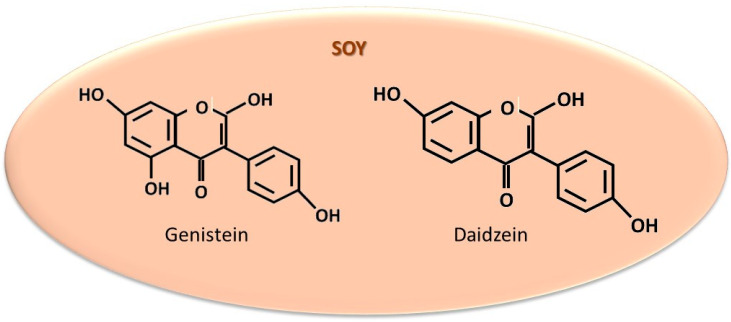
The most significant structures identified in soy, studied for estrogen-like action.

Soy contains isoflavones, such as genistein and daidzein (Figure 5), which possess a structure similar to 17-β-estradiol, enabling them to activate ER [247,248]. Bennetau-Pelissero C [249] emphasized that genistein and daidzein may promote the proliferation of ER + BC cells. Moreover, other authors [250] have suggested that these soy-derived isoflavones can reduce the overall efficacy of AIs. Conversely, an in vitro study demonstrated that genistein in combination with exemestane enhances the antiproliferative effect in AI-sensitive MCF-7 and AI-resistant LTED BC cell cultures. These effects were not observed for letrozole and anastrozole. The authors attributed this finding to the fact that genistein, unlike estradiol, binds with an affinity of 87% to ERβ and only 4% to ERα [67]. Several studies found no association between soy and genistein consumption and increased BC risk [251,252], particularly at moderate intakes of 25–25 mg/day [253]. However, the risk appeared to increase with each additional 30 g/day [254] increment. Other studies have established an inverse correlation between the consumption of soy products and BC risk [247,255,256]. This is attributed to the finding that BC cell proliferation is dependent on ERα activation, whereas ERβ activation suppresses the stimulatory effect mediated by ERα [253,257]. Larger clinical trials in human subjects are warranted to eliminate these discrepancies and to establish a beneficial or neutral quantitative range for patients with ER + BC. Additional research is necessary to resolve these controversies and to accurately identify the specific conditions within the human body under which both types of events occur.

#### 5.1.2. Milk

Milk is a food source that potentially contains estrogen-like compounds capable of binding to ER. It is well established that milk contains estrone sulfate and estradiol, both of which remain stable during heat processing [240]. Their effects on ERα in BC are potentiated by insulin-like growth factor 1 (IGF-1), a major activator of the mTORC-1 pathway [258,259]. In turn, mTORC-1 activates the transcriptional activity of ERα [258]. Another component found in bovine milk, zearalenone, is also capable of activating both ERα and ERβ [260]. In addition to these factors, the role of bisphenol—present in UHT milk packaging—in BC pathogenesis should not be overlooked [261,262]. Given the controversy surrounding the association between high dairy intake and BC development [258,263,264,265], it is recommended that patients avoid bovine milk and dairy products following an ER + BC diagnosis. The rationale for this recommendation includes both the potential interference with AIs and the pro-proliferative role associated with this type of food. Nevertheless, conflicting evidence exists; one study highlights the benefits of dairy-derived peptides regarding their antiproliferative and anti-invasive effects on BC [266], while another study found no significant association between dairy consumption and BC [267]. Another study associated fermented dairy products with a protective effect in women with ER + BC, whereas high-fat dairy products did not exhibit this benefit [268]. In another investigation, milk consumption was associated with a reduced risk of both pre- and postmenopausal BC [269]. This finding is supported by other authors who emphasize that moderate consumption of approximately two servings/day may be beneficial [270]. Given these contradictory data and conclusions, further analysis involving larger cohorts of patients with BC are necessary to clarify these findings. Furthermore, the intake of chemical contaminants and c toxic compound found in dairy products—which could play a pivotal role in malignant transformation and progression—should not be overlooked.

#### 5.1.3. Sesame Seeds and Oil

**Figure 6:**
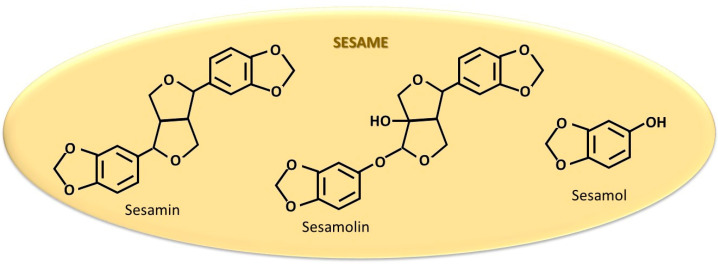
The most studied compounds in sesame for their estrogen-like action.

Sesame seeds and sesame oil contain sesamin, sesamolin, sesamol, sterols (Figure 6), and other biologically active compounds with known antiproliferative and anti-metastatic effects [271,272]. Among these, sesamin is capable of activating ERα [273]. Thus, sesame seeds and oil contribute to the increase in estrogen concentration and aromatase activity in ovariectomized Sprague–Dawley rats [274]. This effect is mediated by the activation of steroidogenesis influenced by the PI3k/protein kinase A or mitogen-activated protein kinase (MAP)/extracellular signal-regulated kinase-2 signaling pathways [274,275]. A review study [276] highlights the effects of sesamin, identified in other seed species. According to the authors, sesamin does not influence aromatase activity but activates ERα and ERβ through estrogen-like action. Another report describes the antiproliferative effect of sesamin on MCF-7 and MDA-MB-231 cell lines [277]. Furthermore, sesamol has been shown to exert a protective effect against lung injury induced as an adverse effect of cyclophosphamide, a chemotherapy agent usually used in BC treatment [278]. Although several studies suggest that sesamin—in combination with other compounds from sesame seeds and oil (sesamol, sesamolin, campesterol, and stigmasterol) and sunflower seeds and oil (quercetin, kaempferol, luteolin, apigenin, CA, and coumaric acid)—may prevent BC development [279], its role in ER + BC remains controversial. Based on these conflicting findings, further research is warranted to elucidate the conditions under which ERα is activated and to determine when sesame seeds and oil provide therapeutic benefits.

In general, ER + BC patients undergoing treatment with AIs are advised to avoid foods, dietary supplements, and plants containing estrogen-like compounds and exercise caution. This practice should occasionally be questioned, as it is largely based on theoretical considerations due to a lack of sufficient clinical studies to justify it. Occasionally, for foods of natural origin, simply avoiding the parts containing substances with estrogenic activity may be a sufficient preventive measure. For example, while the pulp and peel of PG exhibit antiproliferative properties mentioned above, the seeds are rich in sterols and steroids including stigmasterol, β-sitosterol, camesterol, cholesterol, 17-α-estradiol, estrone, testosterone, and estriol [185,280]. In this case, for patients with ER + BC, it is recommended to consume only the pulp and peel, avoiding the seeds or food supplements derived from them. Future investigations will elucidate whether these concerns are justified.

### 5.2. Biologically Active Compounds That Can Interfere Chemically or Pharmacokinetically with Aromatase Inhibitors

Exemestane is primarily metabolized by CYP3A4 into its inactive metabolite 6-hydroxymethylexemestane with only a small fraction being processed by CYP4A11/CYP1A [281]. For letrozole, the inactive carbinol metabolite is produced via CYP2A6 and CYP3A4/5 [282], whereas anastrozole is hydroxylated by CYP3A4 (and, to a lesser extent, by CYP3A5) followed by N-dealkylation [283].

Certain compounds can interfere with the metabolism of AIs either by inhibiting them, which increases toxicity, or inducing them, thereby accelerating metabolism and potentially reducing efficacy. A well-known inhibitor of CYP3A4 is grapefruit juice, which can prolong the effects and increase the incidence of adverse reactions for drugs metabolized by this enzymatic system [284]. On the other hand, certain foods and herbs act as CYP3A4 inducers. Among these, the most well-known is *Hypericum perforatum* [285,286]. Other vegetables such as broccoli, Brussels sprouts, and cauliflower should also be mentioned, as they are rich in glucosinolates that generate indole-3-carbinol and isothiocyanates, particularly sulforaphane. These effects become apparent only after 5–7 days of sustained ingestion [287], an aspect frequency that is less common in modern dietary habits. Furthermore, it appears that 1,1-Bis-3-indolyl methane, a metabolite generated by dimerization of indole-3-carbinol, binds to the aryl hydrocarbon receptor and can reduce tumor growth in ER + BC patients [288]. Moreover, similar effects were observed when indole-3-carbinol and luteolin were combined, as they downregulate ERα and the CDK 4/6 pathway [289]. Comprehensive investigations into the plasma concentrations of AIs in the presence or absence of natural products, inducers, or inhibitors, have not been conducted; consequently, these interactions at the CYP enzyme level remain largely theoretical [290,291]. Nevertheless, some studies advise against combining AIs with *Hypericum perforatum* [292] or grapefruit [293]. While these pharmacokinetic interactions warrant further studies across diverse patient cohorts and combinations, they are rarely encountered, except among regular consumers of *Hypericum perforatum* tea.

**Figure 7:**
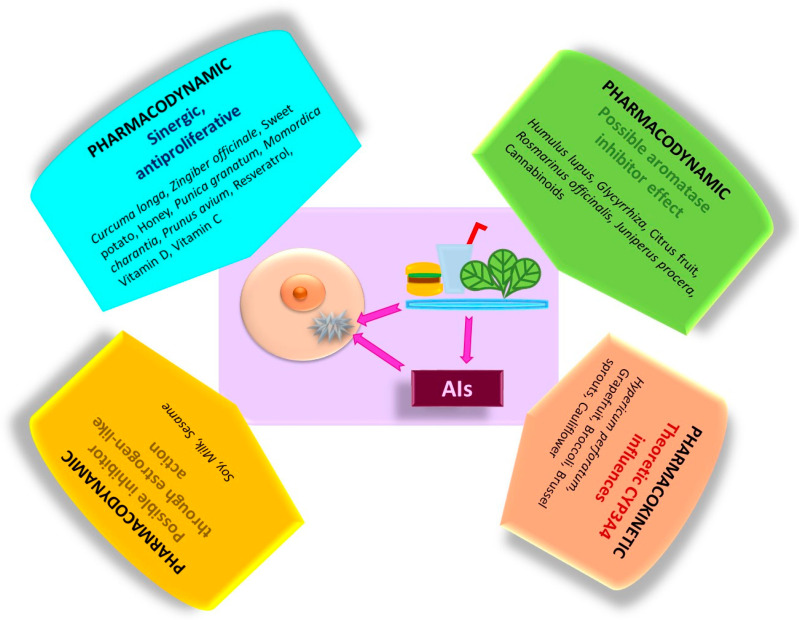
Schematic summary presentation of the main pharmacodynamic and pharmacokinetic effects produced by different natural compounds on AIs and BC.

A summary of biologically active compounds and their effects on BC is displayed in Table 2. Figure 7 illustrates the natural sources of certain substances based on the type of interaction they may produce.

## 6. Conclusions

Patients with ER + BC undergoing AI treatment should consider their dietary choices to optimize therapeutic efficacy. Certain natural products and dietary supplements derived from licorice, rosemary, juniper, cannabis, and citrus fruits may act as competitive aromatase inhibitors. Consequently, their consumption must be carefully monitored and adjusted, as they could potentially interfere with AI therapy. Other nutrients and foods—including honey, ginger, turmeric, sweet potatoes, pomegranates, bitter melon, dark sweet cherries, and resveratrol—are recommended for oncological patients due to their antiproliferative properties demonstrated in both in vivo and in vitro investigations. Patients with ER + BC frequently take vitamins D and C to mitigate certain adverse effects of AIs. Conversely, some natural compounds exhibit estrogen-like activity (soy, cow’s milk, seeds, and sesame oil), raising controversy regarding their potential to reduce the efficacy of AI therapy. Precautions are necessary when combining certain vegetables that may interact pharmacokinetically from a theoretical point of view by interfering with CYP systems. These interferences can occur through enzymatic inhibition (e.g., grapefruit juice) or enzymatic induction (e.g., *Hypericum perforatum*, broccoli, Brussels sprouts, or cauliflower) of AI metabolism. Although these aspects may not seem significant at first glance, they can influence the overall therapeutic response in the long term. Therefore, the treating physician has an obligation to inform patients with ER + BC about which foods are recommended, which should be avoided, and which require special precautions during AI treatment.

The conclusions in this narrative review are subject to certain limitations. Specifically, the properties of biologically active compounds described herein were identified in preclinical investigations including in silico, in vitro, and in vivo animal models. The exact data are unavailable regarding the actual effects on living human tissue or the concentrations these biologically active compounds reached within BC tissue of patients. Furthermore, certain conflicting results regarding these substances are discussed, emphasizing that definitive conclusions can only be drawn within the specific experimental conditions under which they were carried out.

Further research involving larger patient cohorts is warranted. Current clinical trials investigating the associations between anticancer agents and specific foods, active principles or food supplements are often conducted over limited periods, leaving a knowledge gap regarding long-term outcomes. The mechanisms influenced by specific natural substances could serves as starting points for the synthesis and modulation of anticancer drugs. These developments may significantly contribute to the future eradication of BC and cancer in general. The bioactive principles and natural compounds discussed herein provide a basis for identifying the conditions under which, reach therapeutically effective concentrations can be reached within the target tissue. This is particularly relevant in the context of a varied diet, rather than the consumption of a single food item. For nutritional products purchased from retail chains, the presence of preservatives, additives, and other substances used in the manufacturing process is a critical factor. These additives could potentially alter the results observed in studies where only purified compounds of controlled origin were used. Investigations in this area are currently lacking, yet they could be exploited to elucidate the aforementioned controversies and the bridge between laboratory findings and clinical outcomes in real-world patients.

## Figures and Tables

**Table 1:** Recent clinical research showing the current context of using natural compounds in BC patients treated with AIs.

Population/Subjects	Study Type	Intervention	Outcome	References
Women with stage 0–3 hormone-receptor-positive breast cancer, exhibiting AI musculoskeletal manifestations	Phase 2 clinical trial	CBD (Epidiolex)	CBD treatment has good tolerability and safety, improving joint paint	[65]
Postmenopausal women with BC manifesting AI-induced arthralgia	Randomized, placebo-controlled, double-blind pilot trial	Nanoemulsion curcumin versus placebo	This pilot clinical trial showed that nanoformulations of curcumin is a viable option for future studies	[66]
Estrogen-receptor-positive breast cancer cell line	Preclinical, in vitro	Genistein, a phytoestrogen found in soybean with third-generation AIs	Genistein increased the antiproliferative activity of exemestane	[67]
Breast cancer cell lines (MCF-7) and colon cancer lines (COLO 320)	Preclinical, observational, in vitro study	Quercitine, a flavonol compound in combination with anastrozole and capecitabine	This combination might accelerate cell death, control cell growth, stop the cell cycle, and induce mitochondrial depolarization.	[68]
Aromatase inhibitor (Letrozole)- resistant, hormone-dependent breast cancer cell lines (T47DaromLR)	Preclinical, in vitro	Glyceollin, a soy-derivative phytoalexin	Glyceollin reduced proliferation on breast cancer cells.	[69]
Human BC cell lines (MCF-7)	Preclinical, in vitro and in silico	Phytochemical compounds isolated from Carica Papaya leaves	Cytotoxic effect on MCF-7 cell lines and potent aromatase inhibition	[70]

**Table 2:** Biologically active compounds and their potential action on BC.

Natural Compound	Potentially Beneficial	Neutral/Uncertain	Potentially Harmful
*Humulus lupulus*	6-prenylnaringenin, xanthohumol [77]	-	6-prenylnaringenin, 8-prenylnaringenin [80,81]
*Glycyrrhiza*	Liquiritigenin [72,73,74,75]	-	-
Citrus fruits and peel	Naringenin, quercetin, naringin, nobiletin [82,83,84]	Nobiletin + letrozole [84]	-
*Rosmarinus officinalis*	Ursolic acid [94,95], kaempferol [94]	-	-
*Juniperus procera*	2-imino-6-nitro-2H-1-benzopyran-3-carbothiamide [104], juniperolide, kaurenoic acid, isocupressic acid [105]	-	-
*Cannabinoids*	CBD [110,111,112,113,114,115,116]; CBD + exemestane [117]	CBD + letrozole [117]; CBD + anastrozole [117]	-
*Curcuma longa*	Curcumin [139,140,141,142]	-	-
*Zingiber officinale*	Gingerol [146,149], gingerone [147], shogaol [148,151,152]	-	-
Honey	Caffeic acid, chrysin [165,166,167], pinocembrin [168]	-	-
Sweet potato	PPLs [176,179,181], anthocyans [182,183]	-	-
*Punica granatum*	Peel and juice: punicalagin, punicalin [185,194,196]	-	Seeds: stigmasterol, β-sitosterol, camesterol, cholesterol, 17-α-estradiol, estrone, testosterone, estriol [185,280]
*Momordica charantia*	kaempferol, quercetin, momordicoside K [199]	-	-
*Prunus avium*	Anthocyans [213,214,215,216,217]	-	-
Resveratrol	Resveratrol [227,228,229,230,231,232,233,234]	-	-
Vitamin D	1,25-dihydroxyvitamin D3, 1,24-dihydroxyvitamin D3 + AIs [235,236,237]	-	-
Vitamin C	Vitamin C [243,244,245,246]	-	-
Soy	Genistein [247,255,256]; genistein + exemestane [67]	Genistein [251,252,253]; genistein + letrozole, genistein + anastrozole [67]	Genistein, daidzein [249,250]
Milk	Dairy-derived peptides [266], dairy products [269,270]	Zealerenone [259], peptides [267]	Estrone sulfate [258,259], bisfenols [261,262], high-fat dairy products [268]
Sesame	Sesamin [258], sesamol [259]	Sesamin [276]	Sesamin [273], sesame seeds and oil [274]
*Hypericum perforatum*	-	*Hypericum perforatum* [285,286,292]	-
Grapefruit	-	Grapefruit [284,293]	-

## Data Availability

Not applicable.

## Abbreviations

The following abbreviations are used in this manuscript:

AI	aromatase inhibitor
AkT	protein kinase B
AMPK	AMP-activated protein kinase
BC	breast cancer
CA	chlorogenic acid
CBD	cannabidiol
CDK	cyclin-dependent kinases
DFS	disease-free survival
EGFR	epidermal growth factor receptor
ER	estrogen receptor
ESR 1	estrogen receptor gene 1
HDAC	histone deacetylases
HHL	Hops of *Humulus lupulus*
IGF-1	insulin-like growth factor 1
JAK	Janus kinase
JP	*Juniperus procera*
MAP	mitogen-activated protein kinase
MC	*Momordica charantia*
mTOR	mammalian target of rapamycin
mTORC-1	mechanistic target of rapamicyn complex 1
NF-kB	nuclear factor-kB
OFC	ovarian function suppression
OS	overall survival
PA	*Prunus avium*
PG	*Punica granatum*
PI3k	phosphatidylinositol-3 kinase
PPARγ	peroxisome proliferator-activated receptor γ
PPLs	lipid soluble polyphenols
PTM	posttranslational modifications
RO	*Rosmarinus officinalis*
SP	sweet potato
STAT	signal transducer and activator of transcription
VEGF	vascular endothelial growth factor
